# AMP-activated protein kinase regulates L-arginine mediated cellular responses

**DOI:** 10.1186/1743-7075-10-40

**Published:** 2013-05-29

**Authors:** Srinidi Mohan, Harsh Patel, Jorge Bolinaga1, Nathania Soekamto

**Affiliations:** 1 Department of Pharmaceutical Sciences, College of Pharmacy, University of New England, 716 Stevens Avenue, Portland, ME 04103, USA

**Keywords:** L-Arginine, AMPK, Nitric oxide, Superoxide, Peroxynitrite, Endothelial nitric oxide synthase

## Abstract

**Background:**

Our prior study revealed the loss in short-term L-Arginine (ARG) therapeutic efficacy after continuous exposure; resulting in tolerance development, mediated by endothelial nitric oxide synthase (eNOS) down-regulation, secondary to oxidative stress and induced glucose accumulation. However, the potential factor regulating ARG cellular response is presently unknown.

**Method:**

Human umbilical vein endothelial cells were incubated with 100 μM ARG for 2 h in buffer (short-term or acute), or for 7 days in culture medium and challenged for 2 h in buffer (continuous or chronic), in the presence or absence of other agents. eNOS activity was determined by analyzing cellular nitrite/nitrate (NO_2_^–^/NO_3_^–^), and AMP-activated protein kinase (AMPK) activity was assayed using SAMS peptide. ^13^C_6_ glucose was added to medium to measure glucose uptake during cellular treatments, which were determined by LC-MS/MS. Cellular glucose was identified by o-toluidine method. Superoxide (O_2_^•–^) was identified by EPR-spin-trap, and peroxynitrite (ONOO^–^) was measured by flow-cytometer using aminophenyl fluorescein dye.

**Results:**

Short-term incubation of cells with 100 μM ARG in the presence or absence of 30 μM L-N^G^-Nitroarginine methyl ester (L-NAME) or 30 μM AMPK inhibitor (compound C, CMP-C) increased cellular oxidative stress and overall glucose accumulation with no variation in glucose transporter-1 (GLUT-1), or AMPK activity from control. The increase in total NO_2_^–^/NO_3_^–^ after 2 h 100 μM ARG exposure, was suppressed in cells co-incubated with 30 μM CMP-C or L-NAME. Long-term exposure of ARG with or without CMP-C or L-NAME suppressed NO_2_^–^/NO_3_^–^, glucose uptake, GLUT-1, AMPK expression and activity below control, and increased overall cellular glucose, O_2_^•–^ and ONOO^–^. Gluconeogenesis inhibition with 30 μM 5-Chloro-2-N-2,5-dichlorobenzenesulfonamido-benzoxazole (CDB) during ARG exposure for 2 h maintained overall cellular glucose to control, but increased cellular glucose uptake. Continuous co-incubation with CDB and ARG increased NO_2_^–^/NO_3_^–^, glucose uptake, GLUT-1, AMPK expression and activity, and maintained overall cellular glucose, O_2_^•–^ and ONOO^–^ to control conditions.

**Conclusion:**

The present study provides the fundamental evidence for AMPK as the primary modulator of ARG cellular responses and for regulating the mode of glucose accumulation during short-term and continuous ARG treatments.

## Background

Use of L-arginine (ARG) as a dietary supplement has gained popularity in the last decade after its role as the endogenous substrate for nitric oxide synthase was identified. In the National Institute of Health (NIH) website, “Medlineplus” [1], the use of ARG in as many as 44 diseases and diagnoses were discussed and categorized according to the strength of scientific evidence supporting its use. In patients with stable angina pectoris, 6 g ARG/day for 3 days increased their exercise tolerance [2], and when supplemented with 2 food bars enriched with ARG per day for 2 weeks improved vascular function, exercise capacity and quality aspects of life [3]. In patients with congestive heart failure, 9 g ARG/day for 7 days prolonged exercise duration [4,5]. In addition, ARG has been found to improve immunity [6-8], in patients under critical care [9,10] and in sickle cell disease [11,12]. As a supplement, ARG has been commercially endorsed by Nobel laureates, Drs. Louis Ignarro and Ferid Murad, who received their award (with Dr. Robert Furchgott), for their work on nitric oxide pharmacology in 1998. Examples of commercial products include N.O. XPLODE®, NiteWorks™ and Arginine Extreme. Thus, the range of diseases that can be potentially benefited by ARG supplementation is therefore quite wide [9,12-16].

Of concern, the long-term effects of ARG supplementation have not been examined extensively. Only two well-conducted clinical studies are available, and both revealed that the short-term therapeutic benefits of ARG are not evident after long-term use. Study conducted by Wilson et al. [17] showed that ARG supplementation (3g/d) for 6 months, in 133 subjects, “did not increase nitric oxide synthesis or improve vascular reactivity”, and “the expected placebo effect observed in studies of functional capacity was attenuated in the ARG-treated group”. These authors characterized their findings as indications for the existence of “ARG tolerance”, because beneficial effects was observed after one month of dosing. In the Vascular Interaction With Age in Myocardial Infarction (VINTAGE MI) trial [18], a total of 153 patients after MI was randomly assigned ARG (goal dose of 3 g tid) or matching placebos for 6 months. The results showed no improvement in vascular stiffness measurements or ejection fraction. Strikingly, 6 patients in the ARG group died during the study period versus none in the placebo group. The authors therefore concluded that ARG “may be associated with high post-infarct mortality”, and stated that ARG “should not be recommended following acute myocardial infarction”, contrary to the beneficial effects shown by the same regiment after one month [4,15]. These potential long-term deleterious effects of ARG supplementation have also been shown in animal studies [19].

The development of ARG tolerance upon chronic dosing thus represents a major hindrance for the use of this important amino acid to benefit patients. By understanding the mechanism(s) associated with ARG tolerance, the positive effects of ARG supplementation can be extended to benefit patients in whom short-term therapeutic effects have already been demonstrated. Our prior study shows that ARG tolerance in endothelial cell cultures may be mediated by eNOS down-regulation, secondary to oxidative stress and induced glucose accumulation [20]. We have shown in our prior study that continuous ARG exposure to stimulate O_2_^•-^ overproduction via electron transport leak from the mitochondria, thereby triggering oxidation of tetrahydrobiopterin (BH_4_) to dihydrobiopterin (BH_2_) [21]. The diminished BH_4_-to-BH_2_ ratio was then found to be the molecular link between oxidative stress and endothelial dysfunction during long-term ARG supplementation [21]. However the fundamental mechanistic factor that controls ARG mediated O_2_^•-^ production that is responsible in initiating the downstream tolerance sparing events, still remains a puzzle.

The AMP-activated protein kinase (AMPK) [22], a protein consisting of three subunits α, β, and γ, is an important energy-sensing/signaling system by which cells sense and decode changes in energy status. All three subunits are required for expression of full AMPK activity [23]. Activation of AMPK is found to be required for the decrease in glucose production and the increase in fatty acid oxidation, as well as in increasing glucose uptake in skeletal muscles. Thus, the overall effect of AMPK activation is to switch off ATP-consuming pathways such as lipogenesis or gluconeogenesis, whereas switching on ATP-producing pathways such as fatty acid and glucose oxidation. Further, an increase in AMPK activity under various physiological and pathological conditions can lead to an increase in NO synthesis by eNOS [24,25]. Conversely, eNOS knockdown mice, or shear stress [26] suppressed AMPK activity in endothelial cells, emphasizing the importance of endogenous NO in AMPK activation and subsequent metabolism of energy substrates.

The present study aims to explore the possibility of regulation on cellular AMPK activation to be fundamentally responsible in modulating the cellular responses during short-term (acute,) and continuous (chronic) ARG exposures.

## Methods

### Supplies and reagents

Human umbilical vein endothelial cells (HUVEC) was purchased from American Type Culture Collection (Manassas, VA) and culture reagents were from Invitrogen (Carlsbad, CA). All culture supplies and chemicals were from Laboratory Product Sales (Rochester, NY) and Sigma-Aldrich (St. Louis, MO) respectively. Commercial kits were used to examine cellular expression levels of eNOS (by Quantikine human eNOS immunoassay kit; R&D Systems, Minneapolis, MN), AMPK (ELISA Kit by Antibiotics-Online, Atlanta, GA), glucose cellular transporter-1 (GLUT-1 ELISA kit, by My-Biosource, San Diego, CA), NO_2_^–^/NO_3_^–^ (by fluorometric assay kit; Cayman Chemicals, Ann Arbor, MI), and glucose accumulation (by Quantikine glucose assay kit; Bioassay Systems, Hayward, CA). Inhibitors of eNOS (30 μM L-N^G^-Nitroarginine methyl ester; L-NAME), AMPK (20 mM Compound C; CMP-C), and gluconeogenesis (30 μM 5-Chloto-2-(n-(2, 5-dichlorobenzenesulfonamide))-benzoxazole; CDB) were purchased from Santa Cruz Biotechnologies (Santa Cruz, CA). The internal standards for LC-MS studies, ^13^C_6_-ARG [as ARG: HCl (U-^13^C_6_, 98%)], D_4_-L-Cirulline [D_4_-CIT (4,4,5,5-D_4_, 96.5%)], and D_7_-Asymmetric di-methylarginine [ADMA: HCl:H_2_O (2,3,3,4,4,5,5-D_7_, 98%)], as well as ^13^C_6_-glucose and ^15^N_4_-ARG, were obtained from Cambridge Isotope Laboratories, Inc (Andover, MA). Deionized water (18 MΩ) was used in all experiments.

### Cell studies

HUVEC were cultured in physiological F-12K medium containing 100 μM ARG and 4.4 mM glucose. For cellular uptake studies to analyze glucose influx, cells were cultured in physiological F-12K medium containing 100 μM ARG, 4.3 mM glucose and 0.1 mM ^13^C_6_-glucose. All culture medium were supplemented with 20% horse serum, 100 U/mL penicillin and 100 μg/mL streptomycin. Cells were maintained in a humidified chamber at 37°C with 5% CO_2_, and passages between 6 and 14 (mean passage number = 9 ± 3) were used in all the experiments. For acute studies, cell culture in 6-well plates were incubated in Locke’s buffer (154 mM NaCl, 5.6 mM KCl, 3.6 mM NaHCO_3_, 2.3 mM CaCl_2_, 4.4 mM D-glucose, 5 mM HEPES, pH 7.4) containing either 100 μM ARG or combinations of 100 μM ARG with or without 30 μM L-NAME, 20 μM CMP-C, or 30 μM CDB for 2 h. Continuous effect was assessed by incubating cultured cells with 100 μM ARG (and/or other agents) in daily refreshed medium consecutively for 7 days, after which cells are washed twice with 1X phosphate buffered saline (PBS), and challenged with Locke’s buffer containing similar treatment conditions, for 2 hr.

After incubation, cells are then detached from the 6-well plates using trypsin-EDTA, centrifuged at 2000 × g for 30 sec at 4°C, and washed twice using 1X PBS. Cell lysis is carried out at 2-8°C, centrifuged at 13,000 × g for 5 min at 4°C, and the resultant supernatant was used to test eNOS expression, to monitor cellular eNOS, AMPK and GLUT1 expression, besides NO_2_^–^/NO_3_^–^, and glucose accumulation according to instructions provided by the respective manufacturer and correction for protein content [27] is applied.

### Superoxide (O_2_^•–^) measurement using EPR Spectroscopy

In spin trapping measurements of oxygen radical (viz O_2_^•–^) generation were performed on 5 × 10^6^ cells/mL in PBS with DMPO at a final concentration of 50 mM. The DMPO (>97% pure) was purchased from Aldrich and further purified by double distillation. EPR spectra were recorded in a quartz flat cell at room temperature with a Bruker ER 300 spectrometer operating at X-band with a 100-kHz modulation frequency and a TM 110 cavity, as described [28]. The microwave frequency and magnetic field were precisely measured using an EIP 575 frequency counter and Bruker ER 035 NMR gauss meter. Quantitation of the free radical signals was performed by comparing the double integral of the observed signal with that of a known concentration of the 2,2,6,6,-tetramethyl-l-piperidinyloxy free radical in aqueous solution as previously described [28].

### Peroxy (ONOO^–^) measurement

Aminophenyl fluorescein (APF) developed by Tetsuo Nagano et. al., [29] and commercially made available by Cell Technology (Mountain View, CA), was used to determine ONOO^–^ in whole cells; as it has little reactivity towards other hydroxyl reactive oxygen species such as: hypochlorite (^-^OCl), singlet oxygen (021), O_2_^-•^, hydrogen peroxide (H_2_O_2_), NO, and alkyl peroxide (RO_2_^•^). Cells, during the final 15 min of their treatment period are exposed to a final individual concentrations of 5 μM APF, incubated in dark at 25°C, washed twice with 1X PBS, followed by flow-cytometer measurement, with excitation and emission wavelengths of 488 and 515 nm respectively. The dye concentrations of 5 μM for APF are carefully selected after experimental optimization that avoided cell death. This methodology was found to be at least 1 fold more sensitive than traditional chemiluminescence measurement of ONOO^–^. This method was also effective in measuring ONOO^–^ in whole viable cellular treatments under physiological conditions.

### AMPK activity

After treatment, cells were immediately washed with 2 ml of ice-cold 1X PBS buffer and scraped with a rubber spatula in lysis buffer (20 mM Tris, pH 7.5, 150 mM NaCl, 1 mM EDTA, 1 mM EGTA, 1% Triton X-100, 2.5 mM sodium pyrophosphate, 1 mM ß-glycerophosphate, 1 mM Na_3_VO_4_, 1 μg/ml leupeptin, 1 mM phenylmethylsulfonyl fluoride). The cell lysates were then sonicated twice for 10 s and then centrifuged at 14,000 × g for 20 min at 4°C. The pellets were discarded and supernatants were assayed for protein concentration (Lowry, 1951 #302). Duplicate tubes from each sample were prepared and were mixed with 500 μl of IP buffer (lysis buffer plus 1 M NaCl and 1 mM dithiothreitol). AMPK was then immunoprecipitated by adding 10 μg of polyclonal antibody against AMPK (Cell Signaling) and 25 μl of protein A-G agarose (Santa Cruz Biotechnology) and incubated at 4°C. After centrifugation (14,000 × g, 1 min), the beads were washed with IP buffer and then twice with 10X reaction buffer (400 mM HEPES, pH 7.4, 800 mM NaCl, 50 mM MgCl2, 1 mM dithiothreitol). The AMPK activity was assayed by adding 50 μl of reaction mixtures, consisting of 5 μl of reaction buffer, 10 μl of SAMS peptide (1 mg/ml), 10 μl of ATP working stock consisting of 0.1 μl of 100 mM ATP, 1 μl of [^32^P] ATP, and 8.9 μl of H_2_0, 25 μl H_2_0, or 25 μl of 400 μM AMP and incubated at 37°C for 10 min. The beads were quickly pelleted and 25 μl of supernatant was spotted onto P81 Whatman paper. The filter papers were then washed 4–5 times with 1% phosphoric acid. After the final wash, the filters were quickly dried and counted in a scintillation counter. The difference between the presence and absence of [^32^P] AMP is calculated as the AMPK activity.

### LC-MS assay

Concentration of ^13^C_6_-glucose in cells and medium were determined by LC-MS/MS assay previously described [30]. Briefly, aliquot of 20 μL (of the cellular lysate sample or the medium) was mixed with internal standards (20 μL of 1μM, ^13^C_6_-ARG, 20 μL of 1 μM D_4_-CIT and 20 μl of 250 nM D_7_ ADMA) and protein was removed by adding of 120 μL of acetonitrile containing 0.5% acetic acid and 0.025% trifluoroacetic acid. After centrifugation at 10,000 × g for 20 min, the supernatant was collected for analysis.

### Statistical analysis

Data are presented as mean ± standard deviation after analyzing 6 independently generated samples for each treatment condition (n = 6), unless otherwise stated. Statistical comparisons among groups were performed using one-way analysis of variance (ANOVA), followed by Fisher’s and Tukey’s post-hoc test procedure (Minitab 16.0). Statistical significance was concluded when p < 0.05.

## Results

### Effect of cellular regulation on eNOS function and oxidative stress

**Figure 1:**
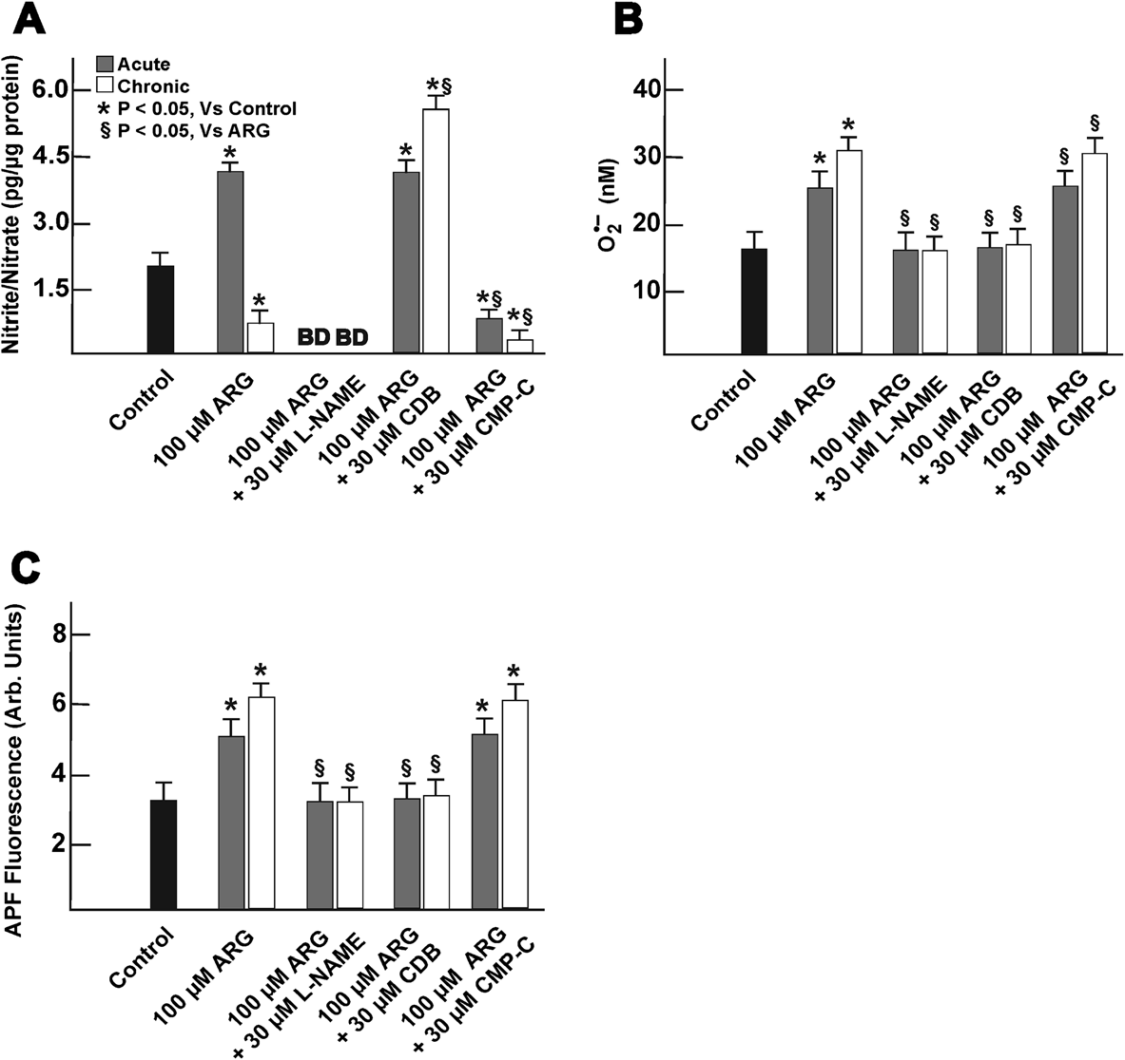
**Comparing the effects of ARG co-incubation with various agents on eNOS function and oxidative stress.** HUVEC were incubated with either 100 μM ARG for 2 h in buffer (short-term or acute exposure), or for 7 days in culture medium and challenged for 2 h in buffer (continuous or chronic exposure), in the presence or absence of other agents. Total NO_2_^–^/NO_3_^–^ assay measurement (**A**) shows the importance in maintaining eNOS and AMPK activity with cellular NO production. The inhibition of AMPK or eNOS function, increased cellular O_2_^•–^ (**B**) and ONOO^–^ (**C**), that were maintained close to control conditions when cellular glucose synthesizing pathway was disrupted. n = 6. *, represents significant variation between control and treatment groups at p < 0.05. §, represents variation in treatment groups from cells treated with 100 μM ARG, at p < 0.05.

HUVEC exposed to 100 μM ARG for a short-term or acute period of 2 h showed significant increase in total nitrite/nitrate (NO_2_^–^/NO_3_^–^) levels from 1.7 ± 0.2 to 4.3 ± 0.3, which were measured as stable end derivative of NO in cells, to determine eNOS activity. The increase in cellular NO_2_^–^/NO_3_^–^ reduced significantly to levels below control conditions in cells chronically or continuously exposed to 100 μM ARG for 7 days and during ARG co-incubation with 30 μM CMP-C, that inhibits AMPK activity (Figure 1A). Short-term or continuous ARG co-incubation of cells with 30 μM L-NAME resulted in reduction in NO_2_^–^/NO_3_^–^ to below detection limit, thus indicating complete inhibition in cellular eNOS activity. Cells treated with ARG in combination with 30 μM CDB showed significant progressive improvement in eNOS function, with increase in NO_2_^–^/NO_3_^–^ that was seen during short-term (4.4 ± 0.4) and continuous (5.7 ± 0.4) treatments.

The increase in NO_2_^–^/NO_3_^–^ after short-term 100 μM ARG exposure was accompanied with at least one-fold increase in both cellular O_2_^•–^ and ONOO^–^, which remained elevated during continuous ARG exposure (Figure 1B and C). Loss in AMPK activity that was observed in cells treated with 100 μM ARG in the presence of 30 μM CMP-C mimicked the elevated O_2_^•–^ and ONOO^–^ levels that were seen during ARG exposure, while cellular co-exposure to either 30 μM L-NAME or CDB during short-term or continuous co-treatment with 100 μM ARG retained the level of O_2_^•–^ and ONOO^–^ to those observed during control conditions.

### Effect of cellular regulation on ^13^C_6_ glucose transport

**Figure 2:**
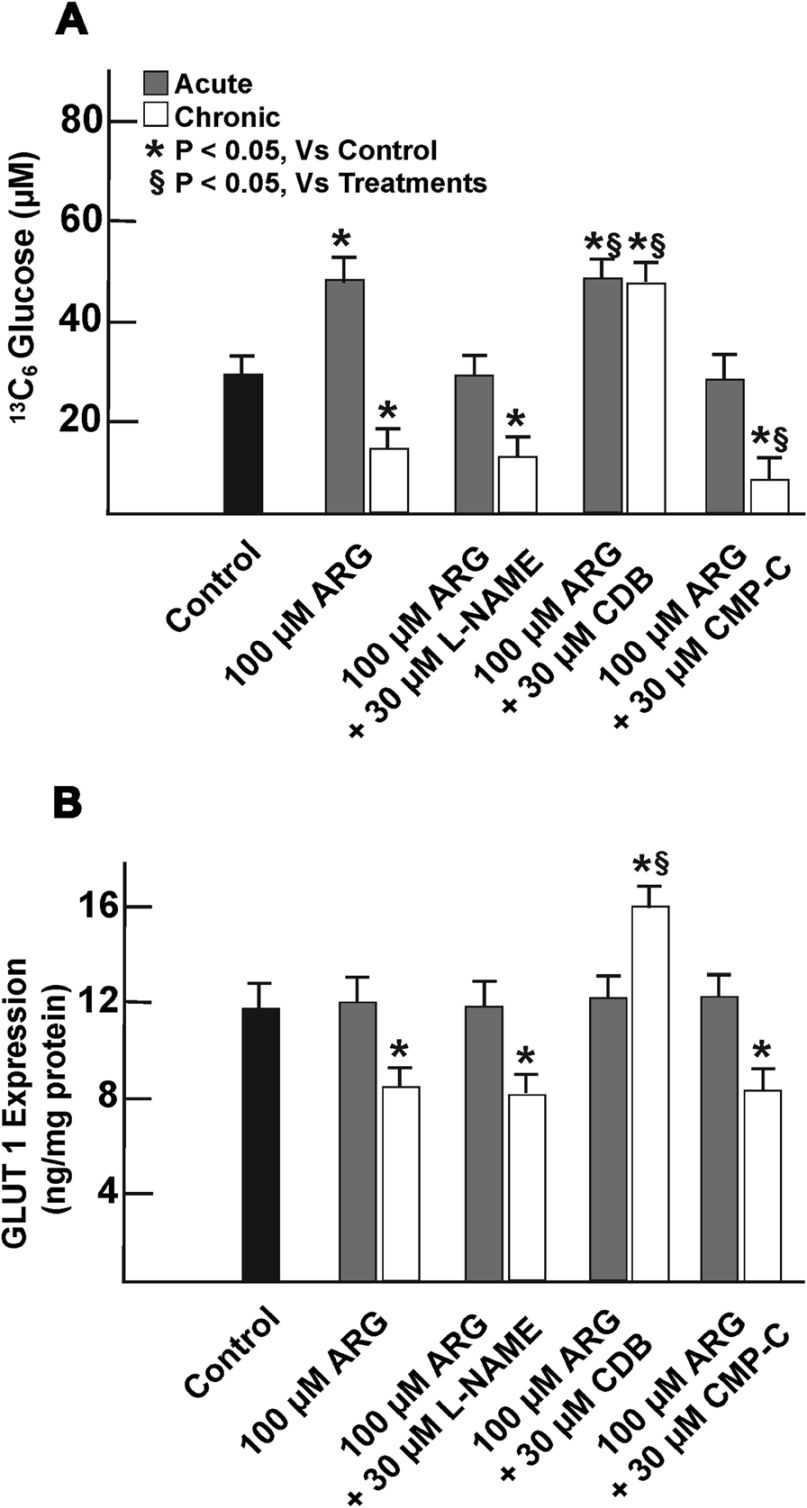
**Analyzing the effect of cellular treatments on **^**13**^**C**_**6 **_**glucose uptake and GLUT-1 expression.** Continuous (or chronic) cellular exposure to 100 μM ARG for 7 days in the presence or absence of 30 μM L-NAME or 30 μM CMP-C decreased ^13^C_6_ glucose uptake from the medium (**A**) and GLUT-1 expression (**B**). Inhibition of cellular accumulation of glucose via gluconeogenesis improved ^13^C_6_ glucose and GLUT-1 expression. n = 6. Acute (or short-term) treatment represents 2 h exposure to cells with various agents. *, represents significant variation between control and treatment groups at p < 0.05. §, represents variation in treatment groups from cells treated with 100 μM ARG, at p < 0.05.

Short-term exposure in cells with 100 μM ARG increased cellular ^13^C_6_ glucose uptake by one-fold, to 49 ± 3.2 μM, which were maintained at levels similar to control conditions (29 ± 1.8) in cells treated for 2 h with 100 μM ARG in the presence of either 30 μM L-NAME or 30 μM CMP-C (Figure 2A). Continuous exposure of cells with ARG alone or in combination with either L-NAME or CMP-C significantly reduced ^13^C_6_ glucose uptake to levels that were at least one-fold below control conditions. Those cells co-incubated with 100 μM ARG and 30 μM CDB maintained the one-fold short-term increase in ^13^C_6_ glucose uptake to 53 ± 4.1 μM during continuous exposure.

The expression level of GLUT-1 protein in HUVEC remained unchanged from control during short-term 2 h treatment of cells with ARG, in the presence or absence of the inhibitory agents for eNOS, AMPK activity or cellular glucose production (Figure 2B). However, at least one-third fold decrease in GLUT-1 protein expression was seen in those cells exposed continuously for 7 days in medium containing 100 μM ARG or when combined with 30 μM of either L-NAME or CMP-C. Continuous exposure of cells with 100 μM ARG in combination with 30 μM CDB for 7 days resulted in one-third fold higher GLUT-1 expression than control.

### Effect of cellular regulation on overall glucose accumulation

**Figure 3:**
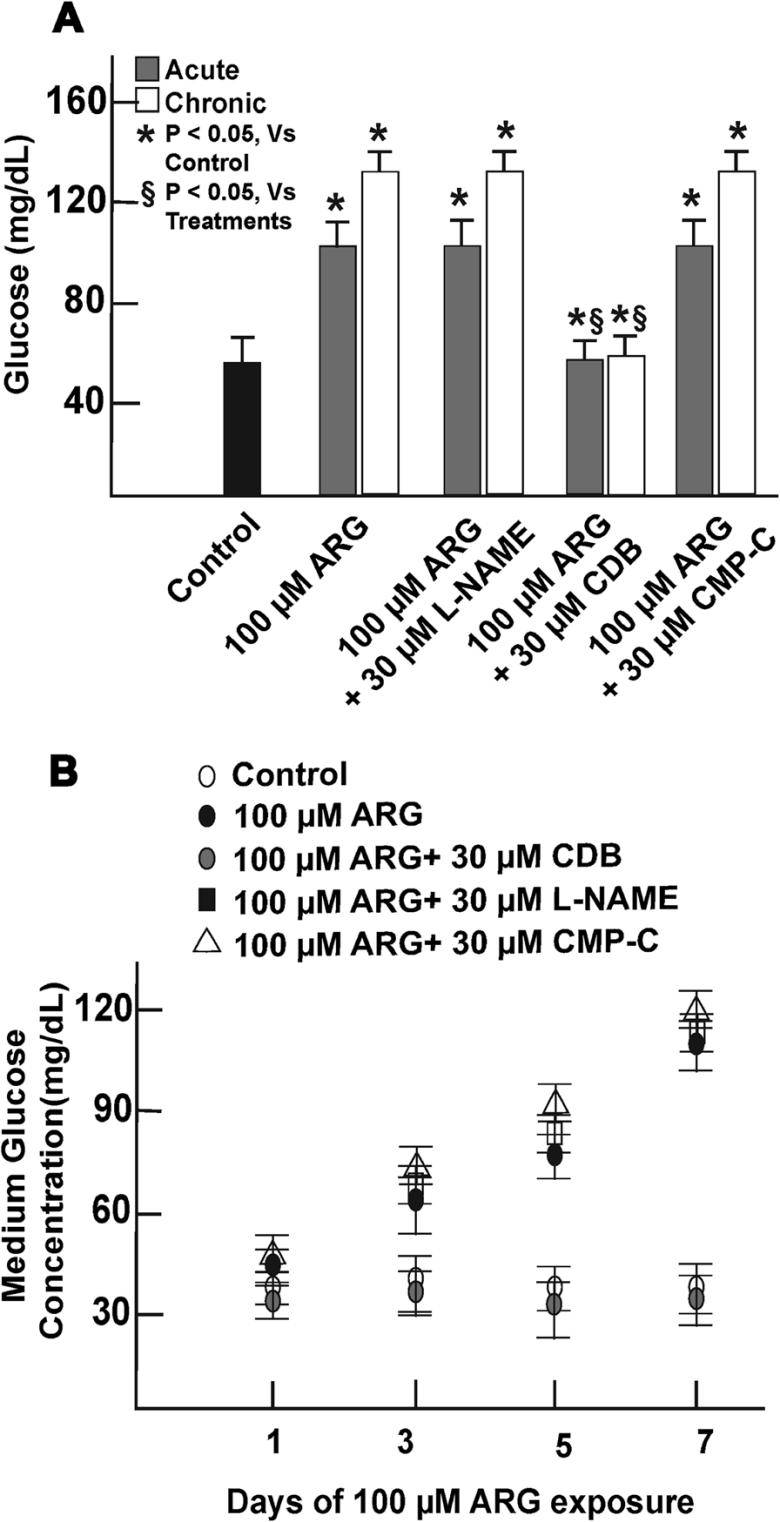
**Measurement of total glucose in cells and medium.** Short-term (acute) or continuous (chronic) cellular exposure to 100 μM ARG in the presence or absence of 30 μM L-NAME or CMP-C resulted in increase in cellular glucose (**A**). A progressive increase of glucose in the cell medium was also observed during ARG continuous treatment of cells for 7 days or when co-incubated with 30 μM L-NAME or CMP-C (**B**). Cells co-incubated with 100 μM ARG and 30 μM CDB, had glucose levels in cells and in the medium at levels similar to control conditions. n = 6. *, represents significant variation between control and treatment groups at p < 0.05. §, represents variation in treatment groups from cells treated with 100 μM ARG, at p < 0.05.

Cells exposed to 100 μM ARG or when co-treated with either 30 μM of either L-NAME or CMP showed at least half-fold increase in overall cellular glucose levels during short-term exposure, that resulted in at least one-fold increase in overall cellular glucose after continuous treatment (Figure 3A). A progressive increase in glucose accumulation in extracellular medium was also observed during continuous exposure to ARG in the presence or absence of L-NAME or CMP-C co-treatment (Figure 3B). Cells incubated with ARG in the presence of 30 μM CDB, maintained cellular and medium glucose levels that resembled control conditions even after continuous exposure.

### AMPK expression and activity during cellular exposure conditions

**Figure 4:**
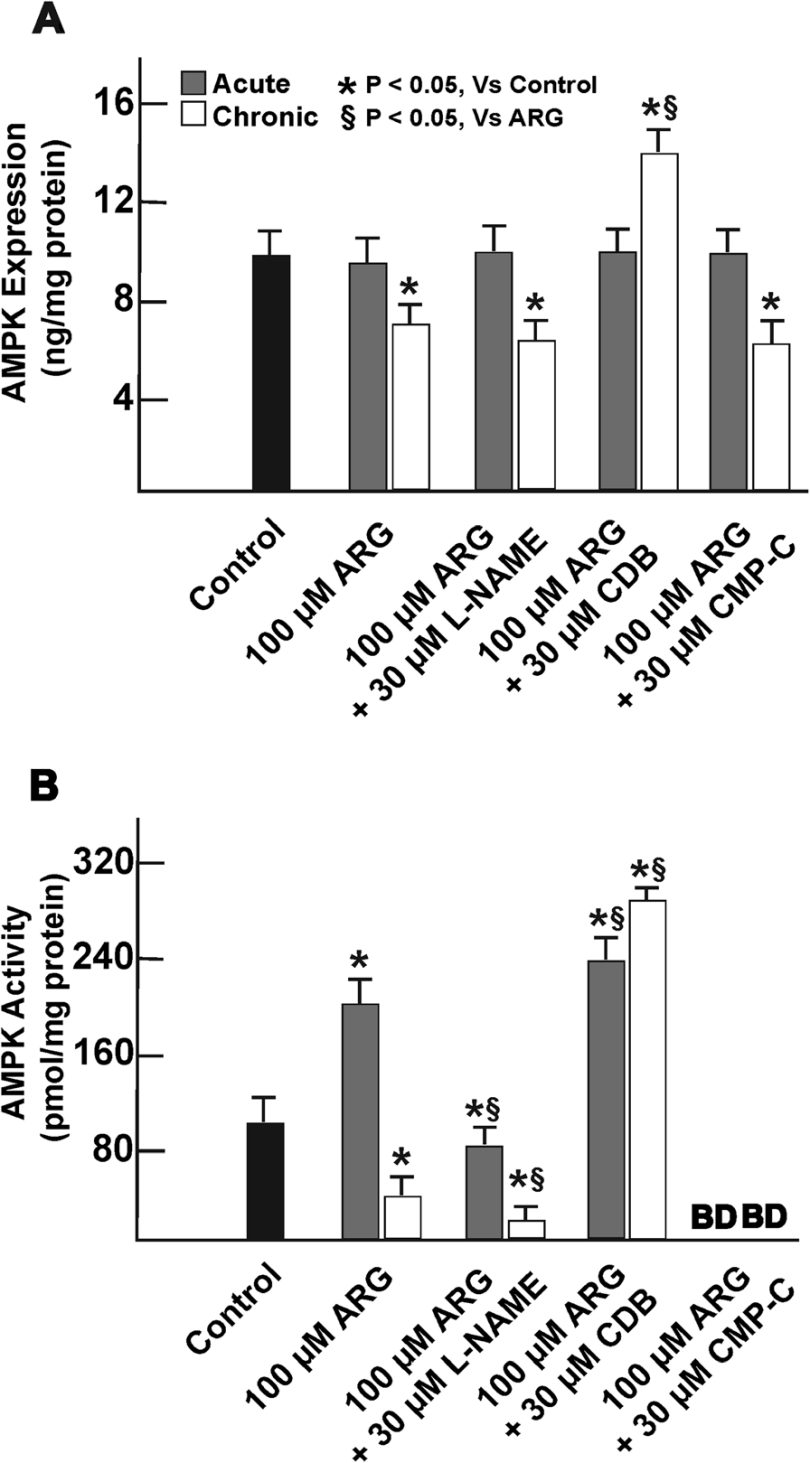
**Measurement of cellular AMPK activity and expression.** AMPK expression decreased only during continuous (or chronic) exposure to 100 μM ARG in the presence or absence of 30 μM CMP-C or L-NAME for 7 days (**A**). Continuous co-incubation of cells with 100 μM ARG and 30 μM CDB for 7 days increased AMPK expression. Cells incubated short-term (or acutely) with 100 μM ARG for 2 h or when continuous co-treated with 100 μM ARG and 30 μM CDB showed increase in their AMPK activity (**B**). Co-incubation of cells with 100 μM ARG and either 30 μM CMP-C or L-NAME suppressed AMPK activity under all treatment conditions. n = 6. *, represents significant variation between control and treatment groups at p < 0.05. §, represents variation in treatment groups from cells treated with 100 μM ARG, at p < 0.05.

Short-term exposure of cells to either 100 μM ARG alone or in combination with 30 μM concentration of L-NAME, CDB or CMP-C, showed no significant variation in AMPK expression levels (Figure 4A). While continuous exposure of cells with 100 μM ARG or in combination with 30 μM L-NAME or CMP-C caused reduction in AMPK expression levels to be at least one-third fold below control, continuous co-incubation of those cells with 100 μM ARG and 30 μM CDB saw at least one-forth fold increase in their overall AMPK expression.

Short-term cellular exposure to 100 μM ARG improved cellular AMPK activity, but experienced at least 1.5 fold reduction in AMPK activity from control during continuous exposure (Figure 4B). Co-incubation of cells with 100 μM ARG and 30 μM L-NAME progressively decreased AMPK activity below control during acute and continuous treatments, and was totally abolished in cells co-treated with 100 μM ARG and 30 μM CMP-C. Short-term and continuous cellular co-exposure to 100 μM ARG and 30 μM CDB saw progressive increase in AMPK activity.

## Discussion

Our study provides the primary evidence to suggest AMPK regulation as the fundamental contributor in modulating ARG response during short-term and continuous supplementation. The therapeutic effect of ARG is based on its ability to act as the endogenous substrate for eNOS in producing NO [31]. Since, endogenous AMPK activation in cells is considered to be vital for eNOS activation via phosphorylation [32], the AMPK-eNOS-NO pathway seems to play an essential role in maintaining vascular function and homeostasis.

The NO generated by eNOS via ARG utilization, is known to be required for the initial activation of AMPK [32], possibly via calmodulin dependent protein kinase kinase (CaMKK) [33,34] or other mechanistic pathways [35,36]. While NO controls AMPK function through activation of guanylyl cyclase, ONOO^–^ that is formed by the reaction between NO and O_2_^•–^, can also impose its regulation on AMPK activation by impairing guanylyl cyclase [37-39]. Thus it can be predicted that the NO-CaMKK-AMPK axis to be predominant in displaying the ARG beneficial effect during short-term exposure, whereas the ONOO^–^-AMPK axis is likely to operate the deleterious effects of continuous ARG exposure. The potential ability for AMPK to act as the fundamental modulator of ARG cellular response during short-term and continuous ARG supplementation is validated in our studies using various inhibitors, whose dosage levels were determined after a dose–response study that showed minimal cell death.

HUVEC exposure for a short period of 2 h to 100 μM ARG, caused a substantial 1.5-fold increase in NO_2_^–^/NO_3_^–^ level that got significantly reduced from control conditions of 1.7 ± 0.2 to 0.5 ± 0.1 pg/μg protein, when cellular AMPK activity was inhibited with 30 μM CMP-C during 100 μM ARG co-treatment. The NO_2_^–^/NO_3_^–^ level were measured in our study (as a stable end-derivatives of NO) instead of cellular NO for determining eNOS activity, due to the short-half life of NO, in the range of few seconds. The decrease in NO_2_^–^/NO_3_^–^ seen during short-term cellular co-incubation for 2 h with 100 μM ARG and 30 μM CMP-C was comparable to those cells continuously exposed to 100 μM ARG alone; and also resulted in concomitant one-fold increase in O_2_^•–^ and ONOO^–^, thereby suggesting the development of oxidative stress under these tested conditions. While the mechanism involved in how AMPK activation is lost during continuous ARG exposure is still under investigation in our lab, the present study provides fundamental evidence suggesting AMPK modulatory to affect eNOS function and cellular oxidative stress during ARG supplementation.

Inhibition of eNOS activity with 30 μM L-NAME during cellular co-incubation with ARG, reduced NO_2_^–^/NO_3_^–^ to below detection limit. The reduction in NO_2_^–^/NO_3_^–^ was also accompanied by decreases seen in AMPK activity level during short-term and continuous exposure, thus suggesting the importance of maintaining eNOS activity, for subsequent activation of AMPK in cells. Although co-incubation of cells with ARG and L-NAME completely blocked eNOS activity and avoided NO generation, an increase in cellular O_2_^•–^ and ONOO^–^ level was not observed, as L-NAME exposure in cells is known to have the ability in controlling both NO and O_2_^•–^ production [40-42]. Thus the use of L-NAME undermines the understanding of ARG response towards cellular oxidative stress under these tested conditions. The use of 30 μM N^G^-monomethyl-L-arginine (L-NMMA) in place of L-NAME abolished eNOS activity in cells when co-incubated with 100 μM ARG, with at least an one-fold increase in O_2_^•–^ and ONOO^–^ to 32 ± 3.4 nM and 5.9 ± 0.1 Arb Units, that was observed from control conditions of 14.8 ± 2.1 nM and 3.1 ± 0.1 Arb Units, respectively. A concomitant decrease in AMPK activity was also observed during 100 μM ARG and 30 μM L-NMMA co-incubation, that were identical to cellular co-treatments with 100 μM ARG and 30 μM L-NAME. These results suggest eNOS function and AMPK activity to be inter-related, but require further evaluation to better delineate their relationship. The use of 30 μM L-NAME or L-NMMA as eNOS inhibitor showed no reduction in eNOS gene expression, when analysed by qPCR (data not shown). Our earlier studies have shown at least a 30% decrease in eNOS protein expression from control in cells continuously exposed with either 100 μM ARG alone or in combination with 30 μM L-NAME or L-NMMA [20], suggesting post-translational modification in these cells.

The use of 30 μM CDB, as inhibitor for gluconeogenesis pathway in combination with ARG showed significant increase in NO_2_^–^/NO_3_^–^, with O_2_^•–^ and ONOO^–^ levels same as control. The 30 μM CDB used in the study did not exhibit any anti-oxidant properties, thereby avoiding the possibility of any artifact in our results. We selected the Fructose-bis-phosphatase (FBPase) inhibitor, CDB, as a probe to modify cellular glucose metabolism, not only because FBPase is a rate-controlling enzyme within the gluconeogenesis pathway, but also because it functions only within the gluconeogenesis pathway, unlike the other two rate limiting enzymes, phosphoenolpyruvate carboxykinase and glucose 6-phosphatase [43,44]. Moreover, adults who are genetically deficient in FBPase activity exhibit relatively normal clinical profiles provided they control their diet and avoid prolonged fasting [45,46].

Primary cell culture studies of various types [47,48], such as the one presented in our study have demonstrate that NO or the intermediates derived from the NO/O_2_^•–^ reaction are not appreciably cytotoxic under the conditions employed [20,21,49]. Under biological conditions where NO has been speculated to be a toxic substance (i.e., cytolytic action of the immune system and ischemia reperfusion), other potent toxic agents such as hydrogen peroxide and superoxide also are present [47,48]. This underscores the importance of investigating the interplay between NO and reactive oxygen species in the mediation of cytotoxicity. Further, the dosage level of the inhibitors used in the present study were implemented after initial dose–response study that avoided cell death as well as anti-oxidant properties, hence eliminating false-positive artifacts in the presented results.

This allowed us to consider the possible relationship between AMPK cellular regulations on glucose uptake from the medium versus endogenous glucose synthesis. To better understand whether AMPK activation is crucial in regulating the mode of cellular glucose accumulation (viz., glucose uptake versus cellular synthesis,) we allowed the cells to be grown in medium containing 0.1 mM ^13^C_6_ glucose, in addition to 4.3 mM D-glucose, resulting in a final glucose concentration of 4.4 mM that is equivalent to physiological conditions. The use of physiological medium condition in our present study helped us in better illustrating the cellular responses to various ARG challenges that were otherwise difficult to measure.

The amount of total glucose accumulation (endogenous glucose + ^13^C_6_ glucose) in cells was analyzed after exposure to 100 μM ARG in the presence or absence of the inhibiting agents for eNOS function, AMPK activity or gluconeogenesis. Those cells subjected to AMPK and eNOS activity inhibition during ARG co-exposure (during short-term and continuous treatment conditions) showed significant increase in overall glucose accumulation with decrease in glucose uptake levels from control. Cellular inhibition of gluconeogenesis pathway with CDB in the presence of ARG increased ^13^C_6_-glucose level similar to those observed during short-term exposure of cells to 100 μM ARG. These short-term increase in ^13^C_6_ glucose uptake level was successfully retained during continuous CDB and ARG co-exposures. The inhibition of gluconeogenesis pathway, also demonstrated a concomitant increase in the expression of cellular GLUT-1. The AMPK expression and activity were also positively improved under these conditions.

These results from this study suggest that ARG mediated short-term therapeutic benefits to be initiated via the activation of AMPK, which stimulates downstream NO release by maintaining eNOS activity and allowing glucose to accumulate only via cellular transport. The dysfunction in AMPK enzyme activity affected eNOS function, decreased glucose uptake from medium, increased cellular glucose synthesis and oxidative stress. All of these events seen during AMPK dysfunction are concomitant with those reported to occur during continuous ARG supplementation [50]. Thus the present study provides the primary evidence of AMPK regulation as the primary modulator for ARG cellular response, based on two time points; 2 h (short-term or acute exposure) and 7 days (continuous or chronic exposure). Multiple time points of 1, 3, and 5 days, need to be evaluated in the future to better resolve the role of AMPK. Further validation is also presently underway to discuss the role of calcium flux and other processes that are involved to better explain the cascade of pharmacological events that regulates AMPK function during ARG supplementation. Better understanding of these pharmacological events will help to inform the design and evaluation of future clinical trials involving ARG.

## Competing interests

The authors declare that they have no competing interests.

## Authors’ contribution

SM performed the O_2_^•–^ measurement by EPR-Spin trap, ONOO^–^ and LC-MS assays. HP, JB, and NS helped in cell treatment, and sample collection. They performed analysis of cellular glucose level, and evaluated the expression levels of eNOS, GLUT-1 and AMPK. They also determined eNOS and AMPK activity and protein content in cell treatments. All authors read and approved the final manuscript.
